# Molecular Features and Metal Ions That Influence 10-23 DNAzyme Activity

**DOI:** 10.3390/molecules25133100

**Published:** 2020-07-07

**Authors:** Hannah Rosenbach, Julian Victor, Manuel Etzkorn, Gerhard Steger, Detlev Riesner, Ingrid Span

**Affiliations:** 1 Institut für Physikalische Biologie, Heinrich-Heine-Universität Düsseldorf, Universitätsstraße 1, 40225 Duesseldorf, Germany; hannah.rosenbach@hhu.de (H.R.); julian.victor@hhu.de (J.V.); manuel.etzkorn@hhu.de (M.E.); steger@uni-duesseldorf.de (G.S.); detlev.riesner@uni-duesseldorf.de (D.R.); 2 Institute of Biological Information Processing (IBI-7: Structural Biochemistry), Forschungszentrum Jülich, Wilhelm-Johnen-Straße, 52428 Jülich, Germany

**Keywords:** catalysis, deoxyribozymes (DNAzymes), gene silencing, metal ion cofactors, RNA hydrolysis

## Abstract

Deoxyribozymes (DNAzymes) with RNA hydrolysis activity have a tremendous potential as gene suppression agents for therapeutic applications. The most extensively studied representative is the 10-23 DNAzyme consisting of a catalytic loop and two substrate binding arms that can be designed to bind and cleave the RNA sequence of interest. The RNA substrate is cleaved between central purine and pyrimidine nucleotides. The activity of this DNAzyme in vitro is considerably higher than in vivo, which was suggested to be related to its divalent cation dependency. Understanding the mechanism of DNAzyme catalysis is hindered by the absence of structural information. Numerous biological studies, however, provide comprehensive insights into the role of particular deoxynucleotides and functional groups in DNAzymes. Here we provide an overview of the thermodynamic properties, the impact of nucleobase modifications within the catalytic loop, and the role of different metal ions in catalysis. We point out features that will be helpful in developing novel strategies for structure determination and to understand the mechanism of the 10-23 DNAzyme. Consideration of these features will enable to develop improved strategies for structure determination and to understand the mechanism of the 10-23 DNAzyme. These insights provide the basis for improving activity in cells and pave the way for developing DNAzyme applications.

## 1. Introduction

**Figure 1:**
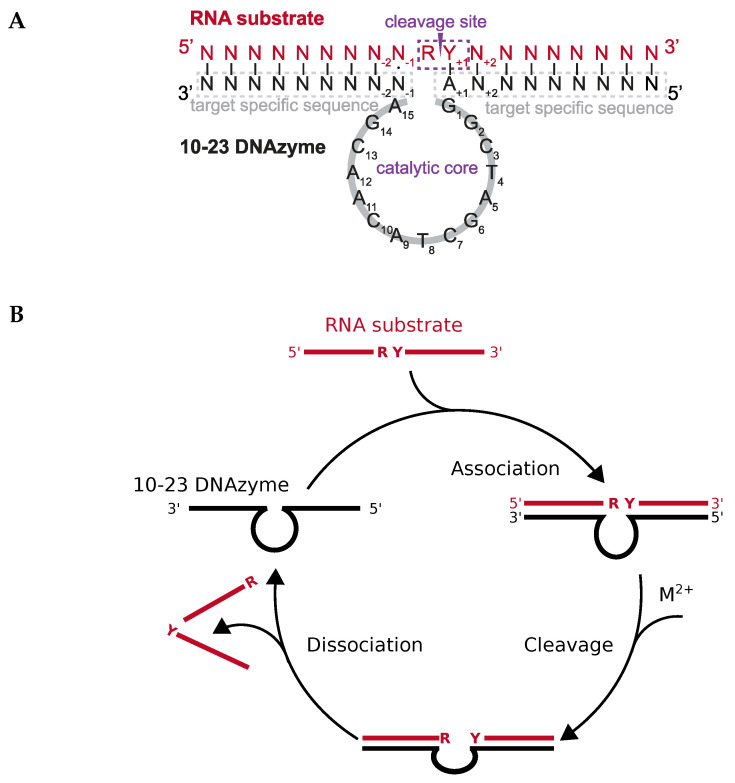
Sequence and reaction scheme of the 10-23 DNAzyme. (**A**) Schematic representation of the 10-23 DNAzyme (indicated in black) with the 15-nt comprising catalytic core bound to the RNA substrate (indicated in red). While in general the binding of the DNAzyme with the RNA substrate occurs via Watson-Crick base pairing (|), nucleotides forming wobble base pairs (⋅) at position −1 lead to enhanced cleavage. (**B**) RNA cleavage performed by the 10-23 DNAzyme under multiple-turnover onditions. The DNAzyme (black) binds its specific RNA substrate (red), cleaves between two central purine (R) and pyrimidine (Y) nucleotides in a manner dependent on divalent cation(s) $M^{2+}$, and dissociates from the cleaved RNA.

Deoxyribozymes or DNAzymes are single-stranded DNA molecules that are capable of catalyzing a variety of chemical reactions, including RNA cleavage [1] and ligation [2], DNA cleavage [3] and ligation [4], the photoreversion of thmyine dimers [5], as well as peptide side chain and backbone modifications [6,7,8,9,10]. The 10-23 DNAzyme is the most extensively studied RNA-cleaving DNAzyme. It was obtained via in vitro selection from a pool of randomized DNA sequences. The 10-23 DNAzyme consists of a catalytic loop of 15 nucleotides that is flanked by two substrate binding arms (Figure 1A). Those arms can be varied in length and sequence in order to allow specific binding to virtually any RNA of interest. After binding, the catalytic loop facilitates cleavage of the RNA substrate between a 5′ central purine and its 3′ neigbouring pyrimidine nucleotide (Figure 1B). The 10-23 DNAzyme has been considered a promising tool to reduce the expression of therapeutically relevant genes on the RNA level [1,11,12,13,14,15,16,17,18,19,20,21]. However, a major obstacle for its in vivo application is the high dependency of the DNAzyme on divalent metal ions. Indeed, recent studies suggest that the 10-23 DNAzyme is catalytically inactive under the conditions inside cells and that visible knockdown effects could be attributed to antisense effects [11,22]. Despite the efforts of several groups to improve catalytic performance by designing DNAzymes that function at lower metal ion concentration, with different metal ion specificity, or even in the complete absence of metal ion cofactors, the activity of the DNAzyme under conditions resembling the cell remains low [23,24,25,26].

In this review, we aim at providing an overview of the structural and functional data on the RNA-cleaving 10-23 DNAzyme. First, we highlight the potential of the 10-23 DNAzyme for therapeutic applications. We then summarize efforts to gain structural insights on DNAzymes utilizing various methods and point out the challenges for this endeavor. We further describe the reaction mechanism with its relevant parameters. In the following sections, we take a closer look at the functional data available on the impact of nucleotide substitutions within the binding arms as well as substitutions and modifications within the catalytic loop. Finally, we outline the influence of various metal ions on activity.

### 1.1. Biological Aspects and Therapeutic Potential of the 10-23 DNAzyme

The ability of the 10-23 DNAzyme to cleave disease-related (messenger or non-coding) RNA makes it particularly attractive as a therapeutic agent. It is highly versatile yet specific. In comparison to the currently more established approaches for post-transcriptional gene silencing, such as the use of RNA interference (RNAi) or antisense deoxyribonucleotides (AS-ODNs), DNAzymes have several remarkable advantages. These advantages include the fact that they—supposedly—are self-sufficient biocatalysts that does not rely on the presence of other biomolecules the way that RNAi relies on argonaut proteins and AS-ODNs on the action of RNase H. These features make DNAzymes also perfect candidates to target RNA-based viruses such as human immunodeficiency viruses (HIV) [27,28,29,30] or corona viruses (CoV): DNAzymes might even be active outside of cells against invading viruses but also inside of cells against viral RNA. In addition, compared to RNA, single-stranded DNA is much more cost-effective and inherently more stable in biological fluids. A growing number of 10-23 DNAzyme variants is currently in preclinical model studies and a small selection in clinical trials focusing on the treatment of basal cell carcinoma and Th2-driven asthma [15,20] (also see e.g., Reference [31] for more detailed review of clinically relevant aspects). While the DNAzyme approach offers a very attractive therapeutic strategy, contradicting observations are found in in vivo experiments, including studies that report on limited catalytic activity under physiological conditions [11,22]. The current situation implies that there is an urgent need to better understand the fundamental processes of DNA-mediated catalysis to enable the design of DNAzymes with improved in vivo activity and unravel the full therapeutic potential of DNAzymes.

### 1.2. Structural Information on DNAzymes

**Figure 2:**
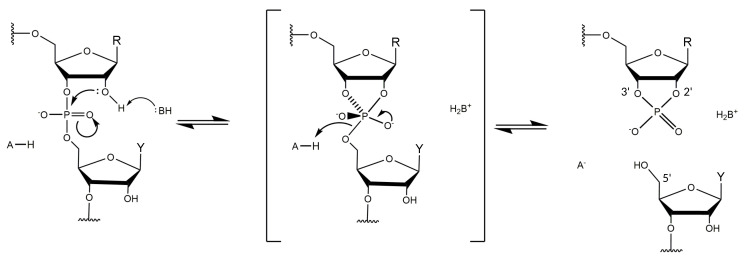
Proposed reaction mechanism of the 10-23 DNAzyme. A proton is abstracted from the 2′-hydroxyl group on the ribose of the unpaired purine nucleotide by an unknown Brønsted base B. The resulting oxyanion functions as a nucleophile that attacks the phosphorus center of the phosphodiesterbond, thus generating a penta-coordinated phosphorane intermediate. The intermediate degrades into two RNA fragments: one fragment that terminates in a 2′-3′-cyclic phosphate and a second fragment that terminates in a 5′-hydroxyl group. R = purine at substrate position 0; Y = pyrimidine at substrate position +1 (Figure 1A). Based on Reference [32].

Structural information on DNAzymes is scarce and the absence of detailed information on the spatial arrangement and the metal coordination sites prevent a deeper understanding of the molecular mechanism of DNAzyme catalysis. Based on its similarity to the hammerhead ribozyme, the RNA-cleaving reaction is believed to involve a transesterification (Figure 2) [32,33]. In the absence of structural data, however, the mechanism of the chemical transformation catalyzed by the 10-23 DNAzyme remains under debate. The first crystal structure reported by Reference [34] with the 10-23 DNAzyme in complex with its RNA target showed the formation of a four-way junction. This arrangement composed of two DNAzyme strands and two RNA substrate molecules is unlikely to represent the catalytically active conformation of the complex. In a follow-up study, Reference [35] presented a combinatorial approach using 81 different DNAzyme:RNA complexes for crystallization screenings. The different biological samples involved different combinations of DNAzymes and RNA substrates with different length, with or without overhangs. Their strategy led to the formation of 40 crystals and data sets with a resolution of up to 2.8 Å. However, the structure solved from these diffraction experiments did not lead to a crystal structure in a catalytically active conformation. A study by Reference [36] found that heat-treatment is effective to prevent formation of the inactive quaternary complex between the two DNAzymes and the two substrates, however, they did not obtain diffracting crystals. Another attempt to obtain high resolution structural information on DNAzymes was reported by Reference [37]. They have crystalized a 52-nucleotide DNA/2′-OMe-RNA oligomer mimicking 10-23 DNAzyme in complex with its substrate and were able to collect data to 1.2 Å resolution. Despite tremendous efforts to solve the phase problem, including direct methods, molecular replacement, and single-wavelength anomalous diffraction of phosphorus atoms, they were not able to derive the phases and obtain an electron density.

All attempts described so far have focused on determining the structure using X-ray crystallography. However, the formation of highly ordered nucleic acid crystals is often challenging due to the surface properties of nucleic acids. While the surfaces of proteins consist of hydrophobic patches that favour intermolecular interactions, the surfaces of nucleic acids are dominated by negatively charged and regularly ordered phosphate groups. Uniform surfaces prevent the formation of distinct interactions between molecules in the crystal lattice and lead to the formation of crystals with poor long-range order. In addition to the challenge of obtaining high quality crystals, solving the phase problem is an additional obstacle in nucleic acid crystallography. The phase problem arises from the loss of phase information during the diffraction experiment. When collecting X-ray diffraction data from a crystal, the intensities of the diffracted waves scattered from a series of planes are measured [38]. From these intensities the amplitudes of the scattered waves are derived. At this point, we lose the phase information, which describes the offset of these waves when we add them together to reconstruct an image of our molecule. For small molecule crystallography determining phases by ab initio approaches is quite common. In protein crystallography phases are derived either by using the atomic coordinates of a structurally similar protein (molecular replacement) or by finding the positions of heavy atoms that are intrinsic to the protein or that have been added (methods such as MIR, MIRAS, SIR, SIRAS, MAD, SAD or combinations of these). Strategies to obtain heavy atom derivatives of RNA crystals [39] have been described as well as co-crystallization approaches with nucleic acid-binding proteins [40,41], but solving the phase problem for nucleic acid data remains nontrivial. The only available high-resolution structural information on DNA catalysts have been obtained for the RNA-ligating DNAzyme 9DB1 and the RNA-cleaving 8-17 DNAzyme. The crystal structure of the DNAzyme 9DB1 has been solved in complex with its two RNA substrates at a resolution of 2.8 Å [42]. The only crystal structure of an RNA-cleaving DNAzyme has been solved in the presence of a DNA instead of the RNA substrate at a resolution of 2.55 Å [40]. However, as the investigated DNA catalyst belongs to the family of the 8-17 DNAzymes the information obtained from this structural study do not allow for any conclusion about the mechanistic aspects of RNA-cleavage performed by the 10-23 DNAzyme. Information about size and shape of the 10-23 DNAzyme in complex with its RNA substrate and the RNA-binding protein U1A has been obtained by small-angle X-ray scattering (SAXS) [43]. Another structural model of the 10-23 DNAzyme in complex with its RNA target has been obtained by molecular dynamics (MD) simulation [44]. However, the latter study was not confirmed by any experimental validation of the presented structure. Attempts to obtain the solution structure of the 10-23 DNAzyme: RNA complex by NMR spectroscopy have not yet been successful [45], but offer a promising alternative strategy. To our knowledge no efforts to solve the structure of DNAzymes utilizing cryogenic electron microscopy (cryo-EM) have been reported so far. The absence of a high-resolution structure of a RNA-cleaving DNAzyme bound to its RNA substrate has hampered the efforts to modify the 10-23 DNAzyme for improved in vivo performance.

### 1.3. Kinetics of DNAzyme-Mediated Catalysis

The catalytic activity of the 10-23 DNAzyme is shown in the scheme of Figure 1B. Shortly, the DNAzyme (Dz) associates with the target RNA to a complex that binds for charge neutralization more cations than the single strands:

(1) $$\begin{array}{cc} \mathrm{Dz}+\mathrm{RNA}+N^{+} & \;⇌\;\mathrm{Dz}:\mathrm{RNA}:N^{+}\;. \end{array}$$

Next; the DNAzyme:RNA complex binds divalent cation(s) to form the catalytically active enzyme:substrate complex that is able to cleave the RNA:

(2) $$\begin{array}{cc} \mathrm{Dz}:\mathrm{RNA}:N^{+}+M^{2+} & \;⇌\;\mathrm{Dz}:\mathrm{RNA}:N^{+}:M^{2+}\;⇌\;\mathrm{Dz}:{\mathrm{RNA}}_{\mathrm{cleaved}}:N^{+}+M^{2+}\;. \end{array}$$

Finally, the enzyme:product complex of DNAzyme and cleaved RNA dissociates into its components

(3) $$\begin{array}{cc} \mathrm{Dz}:{\mathrm{RNA}}_{\mathrm{cleaved}}:N^{+} & \;⇌\;\mathrm{Dz}+{\mathrm{RNA}}_{\mathrm{cleaved}}+N^{+} \end{array}$$

and the DNAzyme is able to enter the catalytic cycle again, starting with (1). These chemical reactions depict a minimal kinetic scheme for catalysis by the 10-23 DNAzyme. In the association step (1), the binding arms of the DNAzyme form basepairs with the target-specific RNA (Figure 1B). The thermodynamic stability of the DNAzyme:RNA complex depends on reactants’ structures, which have to be dissolved at least partially prior to association, on the lengths of the formed helices, the basepair stacking in the helices, and the ionic strength. A well-designed DNAzyme sequence should form neither intramolecular nor intermolecular basepairs with itself; the RNA or at least the target region should also have a low degree of structure [11,46,47]. The stability of the complex increases with increasing length of the helical arms and strongly stacking basepairs [48,49], but a too high stability of the helices inhibits dissociation of the DNAzyme:product complex (3) and thus catalytic turnover [11,50]. Increasing ionic strength overcomes the electrostatic repulsion of the polyelectrolytic nucleic acid single strands (1), leads to higher stability and denaturation temperature of the complex, but may be varied only in vitro while ionic conditions are given in a selected in vivo system. The association reaction (1) is independent from the type of cation; mono- as well as divalent cations facilitate complex formation. In contrast, the catalytic reaction (2) depends on divalent cations M${}^{2+}$; most experimental information is available for Mg${}^{2+}$ or Mn${}^{2+}$ (see below). Notably, divalent cations may substitute for the mentioned monovalent ions in reaction (1), especially in in vitro experiments; on the other hand, high concentrations of divalent cations—especially in combination with slightly basic pH values and/or elevated temperatures—may lead to degradation of RNA [51]. Such high concentrations of divalent cations are quite often used to analyze catalysis of mutated DNAzymes (see below), however, controls for the cation-induced RNA degradation are rarely performed.

The combined reactions (1)–(3) can be summarized and interpreted by a standard Michaelis-Menten equation; that is,

$$\begin{array}{cc} \mathrm{Dz}+\mathrm{RNA} & \overset{k_{f}}{\underset{k_{r}}{⇌}}\mathrm{Dz}:\mathrm{RNA}\overset{k_{\mathrm{cat}}}{\to }\mathrm{Dz}+\mathrm{RNA}{}_{\mathrm{cleaved}} \end{array}$$

with rate constants $k_{f}$ of the forward reaction, $k_{r}$ of the reverse reaction, and $k_{\mathrm{cat}}$ of the catalytic reaction. Multiple-turnover conditions require a DNAzyme concentration much lower than the RNA concentration, a fast dissociation of DNAzyme and cleaved RNA, a sufficiently high $M^{2+}$ concentration, and a low rate of RNA ligation [33]. Under these conditions, the rate of product formation is given by

$$\begin{array}{cc} \frac{d\;[\mathrm{RNA}{}_{\mathrm{cleaved}}]}{d\;t} & =V_{\max}\frac{\left[\mathrm{RNA}\right]}{K_{M}+\left[\mathrm{RNA}\right]} \end{array}$$

with maximum rate

$$\begin{array}{cc} V_{\max} & =k_{\mathrm{cat}}\;\cdot \;\left[{\mathrm{Dz}}_{\mathrm{total}}\right] \end{array}$$

and Michaelis constant

$$\begin{array}{cc} K_{M} & =\frac{k_{r}+k_{\mathrm{cat}}}{k_{f}}\;. \end{array}$$

Most studies described below have not been carried out under reaction conditions at saturating substrate concentrations as required by the Michaelis-Menten model. There is, however, sufficient experimental evidence in the literature (for examples see References [1,11,33,52,53,54,55]) to assume that the Michaelis-Menten model is suitable to describe 10-23 DNAzyme catalysis.

Under single-turnover conditions, that is, DNAzyme is in excess over RNA or the reaction is started by addition of $M^{2+}$ to the preformed Dz:RNA complex, as done in many of the analyses described below, a simple first-order reaction

$$\begin{array}{ccc} \mathrm{Dz}:\mathrm{RNA} & \overset{k_{\mathrm{obs}}}{\underset{⃕}{\to }} & \mathrm{Dz}+{\mathrm{RNA}}_{\mathrm{cleaved}}\\ & M^{2+} & \end{array}$$

is obtained. From the concentration dependence upon time *t*, the rate constant

$$\begin{array}{cc} {\left[{\mathrm{RNA}}_{\mathrm{cleaved}}\right]}_{t} & =\left[\mathrm{Dz}:\mathrm{RNA}\right]\;\cdot \;\left(1-\exp(-k_{\mathrm{obs}}t)\right) \end{array}$$

and yield

(4) $$\begin{array}{cc} Y_{t} & =\frac{{\left[{\mathrm{RNA}}_{\mathrm{cleaved}}\right]}_{t}}{{\left[\mathrm{RNA}\right]}_{t=0}} \end{array}$$

are obtained.

## 2. Impact of Nucleotide Substitutions within the Binding Arms on Catalytic Activity

### DNAzyme Activity Depends Upon Nucleotides Flanking the Cleavage Site

**Figure 3:**
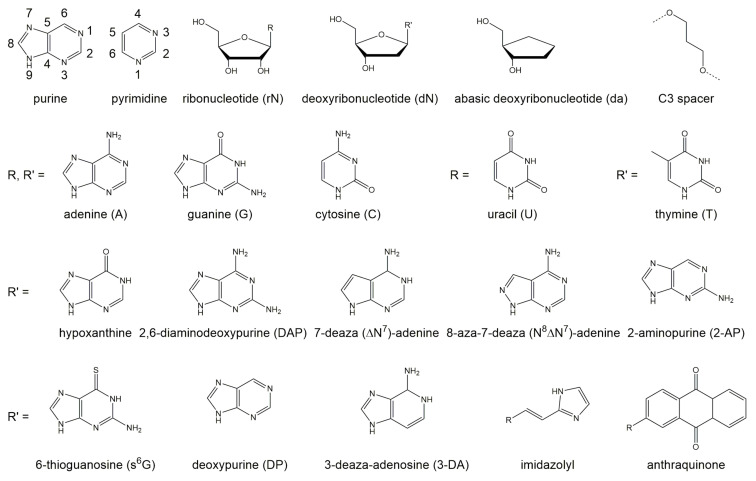
Structures of different nucleotides and C3 spacer used in mutagenesis studies for the 10-23 DNAzyme. The nucleobase hypoxanthine is present in the nucleotide inosine.

The 10-23 DNAzyme cleaves RNA substrates with high nucleotide selectivity at purine-pyrimidine junction sites (rRrY) in the presence of divalent metal ions. The purine nucleotide of the RNA cleavage site remains unpaired while the pyrimidine nucleotide forms a base pair with one of the deoxyribonucleotides of the DNAzyme. The pyrimidine nucleotide of the RNA cleavage site as well as the paired base of the DNAzyme will be referred to as being in position +1 in the following because they are located “downstream” of the actual site of cleavage, that is, between the purine and pyrimidine nucleotide (see Figure 1). In this section we will focus on the (i) impact of base pair mutations at the scissile bond, (ii) the influence of mutations of stacking interactions between the ribonucleotides flanking the cleavage site and the deoxynucleotides at position 1 and 15 of the DNAzyme’s catalytic loop, as well as (iii) the impact of the introduction of modified deoxynucleotides at position 1 of the catalytic loop on the cleavage rate. In 2003, Reference [56] found that the cleavage rates of the DNAzyme for RNA substrates increase in the following order of the cleavage site sequence: rGrU${}_{+1}$= rArU${}_{+1}$ ≥ rGrC${}_{+1}$ ≫ rArC${}_{+1}$. When uridine (rU) is found in the +1 position the nature of the unpaired purine base has no impact on the cleavage rate. However, if cytosine (C) is found in the +1 position the type of the unpaired purine also affects activity. The significantly higher activity of the 10-23 DNAzyme against rRrU substrates over rRrC substrates could be explained by a higher binding strength of the rC${}_{+1}$:dG${}_{+1}$ pairing at position +1 with three hydrogen bonds compared to the relatively weak rU${}_{+1}$:dA${}_{+1}$ pairing with only two hydrogen bonds and altered stacking features. Also, the cleavage rates for substrates with an rRrY junction at the cleavage site are dramatically increased when using DNAzymes with a deoxyinosine (dI) nucleotide instead of the canonical dR counterpart at the +1 position (see Figure 3 for structures of the different nucleotide modifications, which are reviewed in the following; the nucleobase in deoxyinosine is hypoxanthine). The substitution results in two weaker hydrogen bonds rY${}_{+1}$·dI${}_{+1}$ pairing compared to the canonical Watson-Crick base pairing. This effect is particularly striking for substrates with an rArC core sequence, resulting in a rC${}_{+1}$·dI${}_{+1}$ interaction instead of the rC:dG bonding. The opposite effect was observed when using DNAzymes with a diaminopurine (DAP) substitution at position +1 for rRrU substrates. The three hydrogen bond between rU${}_{+1}$ and dDAP${}_{+1}$ pair significantly decreases the cleavage rate of the reaction compared to the weaker rU${}_{+1}$:dA${}_{+1}$ pair.

However, this rate enhancement has only been observed when using substrates with an rArU and not a rGrU cleavage site indicating an influence of the stacking interaction between AU or GU, respectively. This observation goes hand in hand with the one made for substrates with an rRrC core sequence where the cleavage rate was extensively enhanced by a lower binding strength when the unpaired rR nucleotide was an adenosine. In addition to being unpaired, rArY or rGrY cleavage sites require an extended degree of conformational flexibility or lower stacking interaction on the 3′ side (N_+1_) for efficient cleavage rates. This is provided by standard nucleotides when followed by uridine (rU${}_{+1}$:dA${}_{+1}$ pair), whereas a paired dG needs to be replaced by a dI forming a wobble base pair to enhance the activity [56].

Substitution of the dA in an rU${}_{+1}$:dA${}_{+1}$ pair at the cleavage site by 7-deaza (δN${}^{7}$)-deoxyadenine and 8-aza-7-deaza (N${}^{8}\Delta$N${}^{7}$)-deoxyadenine reveal that the nitrogen atom is of equal importance for the cleavage reaction at positions 7 and 8 in the ring system of dA${}_{+1}$, although these atoms do not contribute to thermal stability by forming hydrogen bonds. Deletion of the exocyclic amino group at position 6 or its substitution by bulky groups lead to decreased stability and a lower reaction rate [57]. Table 1 provides an overview of the effect of nucleotide substitutions at position +1 of the DNAzyme (dN${}_{+1}$) and the sequence of nucleotides at the cleavage site of the RNA substrate (paired and unpaired rN${}_{+1}$, respectively) on the reaction rate.

The neighbouring nucleotide rN${}_{-1}$ on the 5′ side of the unpaired purine next to the cleavage site prefers formation of a non-canonical or wobble interaction with the corresponding deoxynucleotide within the DNAzyme. A suitable substitution of this deoxynucleotide in the DNAzyme sequence leads to an increase in the RNA cleavage rate [50,56]. It has been reported that the activity of the DNAzyme against some RNA substrate sequences was improved by base substitutions with reduced interaction strength between the DNAzyme and the substrate. It was shown that the formation of wobble and mispairs immediately 5′ rather than 3′ of the cleavage site are responsible for an enhanced DNAyzme activity [56].

Introduction of an intercalator between the catalytic loop deoxynucleotide dA${}_{15}$ and dN${}_{-1}$ of the DNAzyme binding arm greatly improved the cleavage activity towards an RNA substrate with a rCrG cleavage site compared to the unmodified DNAzyme [58]. The intercalator was attached via an amide bond to a D-threoninol linker that is inserted to the DNA backbone using phosphoramidite chemistry. It was shown, that the catalytic activity was only improved, when the intercalator was introduced to the DNAzyme via D-threoninol, while introduction of methylene between the intercalator and the amide bond significantly lowered the DNAzyme activity. The most enhancing effect on the DNAzyme was detected using an antraquinone intercalator. Furthermore, results obtained with additional intercalators and linkers revealed that planar molecules have an enhancing effect on DNAzyme activity. It was also reported that in the catalytic loop of the 10-23 DNAzyme, dA${}_{15}$ could be exchanged by a nucleobase lacking its 6-amino group to improve the catalytic rate. Furthermore, it was shown that the DNAzyme can be further optimized by adding an extra functional group to the 6-amino group via different C2- or C3-linkers [59]. Other modifications at dA_15_ appeared to show a negative effect on the cleavage activity [60,61].

Furthermore, studies with 2′-*O*-methyl modified nucleotides as well as locked nucleic acid (LNA) substitutions within the binding arms of the 10-23 DNAzyme have shown that modifications that promote an A-form helix of the binding arms enhance the activity of the 10-23 DNAzyme [62,63]. While DNA double helices form B-type helices, the A conformation is common for RNA double helices and has also been reported for DNA-RNA helices [64].

Taken together, a certain degree of flexibility in the base pairing between the substrate nucleotides next to the cleavage site and the corresponding deoxynucleotides in the DNAzyme binding arms is crucial for cleavage activity of the 10-23 DNAzyme. The required conformational freedom might be a hint for a significant rearrangement of the RNA cleavage site towards functional deoxynucleotides in the catalytic loop sequence. This assumption is further supported by the observation that the transition from the catalytic loop to the substrate binding arm, that is, the connection between dA${}_{15}$ and dN${}_{-1}$, can be modified without major influence on the activity, indicating that no rigid conformation is required here.

## 3. Impact of Deoxynucleotide Substitutions and Modifications within the Catalytic Loop on Activity

**Figure 4:**
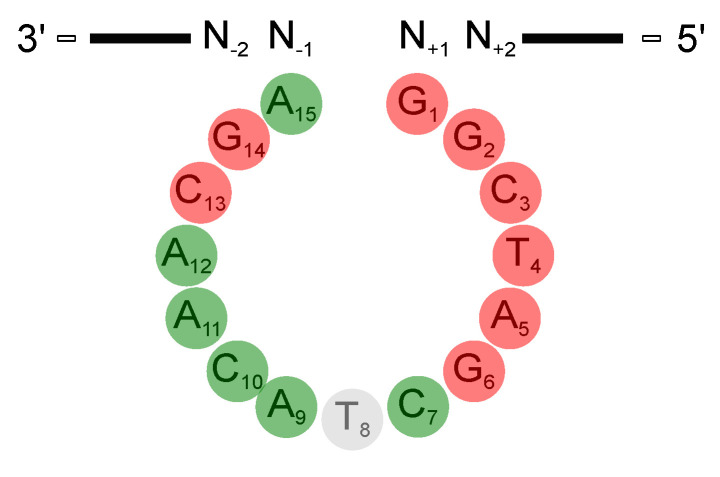
Schematic representation of the 10-23 DNAzyme with emphasis on the deoxynucleotides within the catalytic loop. Modification of the deoxynucleotides 1–6, 13, and 14 (red circles) greatly affect the cleavage rate, whereas exchanges of deoxynucleotides 7–12 and 15 (green circles) only slightly affect the DNAzyme activity. Deletion of the deoxynucleotide dT${}_{8}$ leads to an active 10-23 DNAzyme (grey circle).

The structure of the DNAzyme in complex with its RNA substrate in a catalytically relevant conformation remains unknown. Therefore, much effort has been spend into determining how the deoxynucleotides within the catalytic loop affect DNAzyme function. Several studies have investigated the role of each individual nucleobase [33,57,58,60,61,63,65,66,67,68,69,70], deoxyribose sugars [63,69,71], and the phosphate groups [32,65]. In addition deletion studies provide insights into the relevance of the nucleotides in different positions [72,73,74]. In this section, we will discuss mutational studies with natural and non-natural deoxynucleotides. Moreover, we will outline the impact of substituting individual deoxynucleotides with abasic deoxynucleotides (da) and acyclic C3 spacers for each deoxynucleotide within the catalytic loop region. In contrast to mutations of deoxynucleotides that altered the given structure of the DNAzyme, the introduction of abasic deoxynucleotides or spacers allow for more flexibility of the structure. Figure 4 provides an overview of the role of each nucleotide in the catalytic loop on 10-23 DNAzyme activity at a glace.

### 3.1. The Deoxynucleotides at Position 1 to 6, 13, and 14 Are Crucial for Activity

Reference [70] analyzed the sequence requirements in the catalytic loop of the 10-23 DNAzyme by systematic mutagenesis studies with regard to DNAzyme activity in the presence of ${\mathrm{Mg}}^{2+}$. The reaction of each DNAzyme variant was performed for 20 min at 37 °C in the presence of tenfold molar excess of the DNAzyme over a 19 nucleotide (nt) RNA substrate in the presence of 10 mM MgCl_2_. They used the total yield of the reaction product after 20 min as an indicator of DNAzyme activity. This approach is less labor-intense, however, the results are less reliable as compared to kinetic measurements. Reference [70] found that the deoxynucleotides dG${}_{1}$, dG${}_{2}$, dT${}_{4}$, dG${}_{6}$ and dG${}_{14}$ could not be exchanged independently by any other naturally occurring deoxynucleotide without a complete loss of cleavage activity. Surprisingly, Reference [69] found that the exchange of the deoxynucleotides dG${}_{1}$, dG${}_{2}$ and dT${}_{4}$ by either an abasic deoxynucleotide (da) or a C3 spacer did not lead to a considerably loss of activity. It should be pointed out that the kinetic measurements with the abasic deoxynucleotides and C3 spacers by Reference [69] were performed in the presence of a 100-fold excess of DNAzyme over the substrate in the presence of 200 mM NaCl and 500 mM MgCl${}_{2}$. The introduction of an abasic deoxynucleotide substantially reduces the observed rate constant *k*_obs_ values compared to the unmodified DNAzyme in the following order: da${}_{4}$ > da${}_{2}$ > da${}_{1}$, with the substitution of dT${}_{4}$ showing the largest effect. Mutation of these deoxynucleotides to a C3 spacer does not lower the *k*_obs_ values significantly further compared to the substitution by an abasic deoxynucleotide. In case of da${}_{1}$, da${}_{2}$ and da${}_{4}$, the overall yields after a given time (*Y*${}_{t}$; see (4)) were only slightly reduced when compared to the unmodified DNAzyme, suggesting that the nucleobases may only be necessary for the correct positioning of other nucleotides that are directly involved in the reaction mechanism. Notably, the cleavage assays for the abasic and C3 spacer substitutions were conducted using chimeric DNA/RNA substrates with a single ribonucleotide linkage at the cleavage site [69]. As mentioned previously, DNA:DNA duplexes form B-type helices, while RNA:DNA duplexes form A-type helices. The formation of a different helix type is caused by the different sugar conformation, that is, different sugar puckers, and a different number of base pairs per turn. Additionally, the 2′-OH group on the RNA ribose is not compatible with the deep and narrow minor grove of B-type helices [64,75,76]. In summary, the experiments performed by Reference [69] and Reference [70] point towards a significant importance of the nucleotides dG${}_{1}$, dG${}_{2}$, dT${}_{4}$, dG${}_{6}$ and dG${}_{14}$ under single-turnover conditions.

### 3.2. Exocyclic Functional Groups at Position 6 and 14 Are Crucial for the Activity of the 10-23 DNAzyme

**Figure 5:**
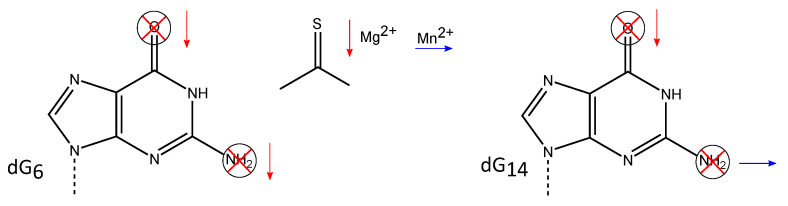
Proposed important exocyclic groups within the 10-23 DNAzyme. The 6-keto group as well as the 2-amino group of both dG${}_{6}$ and dG${}_{14}$ are of functional imporatance for the DNAzyme activity. Deletion of either the 6-keto group or the 2-amino group of dG${}_{6}$ as well as the deletion of the 6-keto group of dG${}_{14}$ lead to an dramatic decrease in catalytic acitivity of the 10-23 DNAzyme as indicated by the red arrows. Substituting the oxygen atom of the 6-keto group in dG${}_{6}$ by a sulfur atom leads to a DNAzyme with significantly reduced cleavage activity in the presence of ${\mathrm{Mg}}^{2+}$ (indicated by a red arrow), but the activity can be rescued in the presence of ${\mathrm{Mn}}^{2+}$ (indicated by a blue arrow). In addition, deletion of the dG${}_{14}$ does not significantly affect the acitivity of the DNAzyme.

The dramatically reduced DNAzyme activity after replacement of dG${}_{14}$ by dI indicates the importance of the 2-amino group. When exchanging the dG${}_{14}$ deoxynucleotide by a 2- aminopurine (2-AP) deoxynucleotide that lacks the 6-keto group of dG, the DNAzyme becomes almost completely inactive [70], emphasizing that both exocyclic functionalities of the guanine base are crucial for the RNA cleavage activity of the 10-23 DNAzyme, although dG${}_{6}$ cannot be exchanged to dA without a complete loss of DNAzyme activity, the deoxynucleotide can be changed to dI with no detectable difference in the cleavage rate. This data suggests that the 6-keto group plays an important role in the cleavage mechanism, whereas the 2-amino group does not appear to be critical for function [70] (Figure 5). This was further confirmed by mutagenesis studies by Reference [65], where modified DNAzymes with a 2-AP substitution as well as a δN${}^{7}$G or a 6-thioguanosine (s${}^{6}$G) replacement at position 6 were assayed for their activity in the presence of either ${\mathrm{Mg}}^{2+}$ or ${\mathrm{Mn}}^{2+}$. The 6-keto group of guanine is lacking in the 2-AP variant, while it is replaced by a thiol group in the s${}^{6}$G mutant. The δN${}^{7}$G lacks the nitrogen atom at position 7 of the guanine base. According to the thio effect based on the Pearson acid-base concept, also termed Hard and Soft Acid and Base (HSAB) model, a non-polarizable hard ${\mathrm{Mg}}^{2+}$ has a lower affinity to the polarizable soft sulfur-containing DNAzyme [77]. As a result, the cleavage rate is lower, whereas a recovery of the cleavage rate, the so-called rescue effect, can be observed when soft ${\mathrm{Mn}}^{2+}$ are used as cofactor instead of ${\mathrm{Mg}}^{2+}$. The different kinetic behavior of the 2-AP and s${}^{6}$G DNAzyme mutants in the presence of either ${\mathrm{Mg}}^{2+}$ or ${\mathrm{Mn}}^{2+}$ [65] confirmed the importance of the 6-keto group proposed by Reference [70] in 2002. The substitution of dG${}_{6}$ by δN${}^{7}$G results in a more than 100-fold loss of activity in the presence of ${\mathrm{Mg}}^{2+}$, which suggests that the N7 nitrogen is involved in the formation of intramolecular hydrogen bonds that are crucial for a functional conformation of the 10-23 DNAzyme [65]. Substitution of the deoxynucleotides dG${}_{6}$ and dG${}_{14}$ by an abasic deoxynucleotide completely abolished the cleavage activity, in accordance with the proposed relevance of the exocyclic functional groups at the nucleobases at positions 6 and 14 [69].

The deoxynucleotides at position 7 to 12 could be replaced by other naturally occurring deoxynucleotides without severe effects. Only the replacement of dC${}_{7}$ to a dA and of dA${}_{9}$ to a dC reduced the cleavage activity by 80% to 90%. While mutation of the deoxynucleotide dC${}_{7}$ to a dG or dI led to a decrease in cleavage activity by more than fourfold, the activity was only slightly affected by substitution to adenosine or thymidine [70]. Substitution by an abasic deoxynucleotide or a C3 spacer significantly reduced the *k*_obs_ value of the cleavage reaction, but only slightly affected the *Y*${}_{t}$ [69]. However, this observation is surprising, since the cleavage reaction was performed for 24 h under single-turnover conditions, in which excess DNAzyme (DNAzyme:substrate ratio 100:1) was used in the presence of 200 mM NaCl and 500 mM MgCl${}_{2}$. Under these conditions complete cleavage of the RNA substrate would be expected. Thus, incomplete cleavage can be a hint for either insufficient association of the uncleaved substrate with the DNAzyme or for complex formation in an inactive conformation. The same is true for dA${}_{9}$, where only the substitution to a dC, an abasic deoxynucleotide or a C3 spacer leads to a significant decrease in the cleavage rates [70,72]. Replacement of dA${}_{11}$ or dA${}_{12}$ by dI did not have a strong effect on the cleavage activity. In case of dC${}_{7}$, dT${}_{8}$ and dC${}_{10}$, the exchange to dI or dG does not abolish DNAzyme activity. Deletion studies performed with a DNAzyme lacking dC${}_{7}$ confirm that this variant still retains a relatively high activity (approx. 60–80%, depending on the substrate sequence) towards a 19 nucleotide RNA substrate [72]. At positions dA${}_{9}$, dA${}_{11}$, and dA${}_{12}$ the exchange of the dA by dI led to a twofold decrease in the cleavage activity, which makes a significant importance of the 6-amino group for the cleavage mechanism at these positions unlikely [70]. Substituting dC${}_{10}$, dA${}_{11}$, or dA${}_{12}$ by abasic deoxynucleotides decreased the *k*_obs_ values by 90%. For the deoxynucleotides dA${}_{12}$ and dA${}_{11}$ no effect on *Y*${}_{t}$ was observed for substitutions with abasic nucleotides and a C3 spacer, while the *Y*${}_{t}$ was reduced by about 50% when changing dC${}_{10}$ for an abasic deoxynucleotide or a C3 spacer. Surprisingly, the mutants with an abasic deoxynucleotide or a C3 spacer at position 8 were found to exhibit a slightly increased $k_{\mathrm{obs}}$ and *Y*${}_{t}$ value compared to the unmodified DNAzyme [69]. Introduction of specific functional groups at position dA${}_{9}$ appear to have an enhancing effect on the DNAzyme activity [59,60,68].

### 3.3. The Exocyclic Amino Groups at dC${}_{3}$ and dC${}_{13}$ Display Additional Functional Importance

The exchange of the nucleotides at positions dC${}_{3}$ and dC${}_{13}$ to dG, dT, or dI drastically decreases DNAzyme activity, whereas a mutation to dA is well tolerated [70]. Substitution of dC${}_{13}$ by an abasic deoxynucleotide completely abolishes activity, in accordance with the importance of the exocyclic 4-amino group. In comparison, total removal of the base at dC${}_{3}$ only reduces the $k_{\mathrm{obs}}$ by 50% and does not affect the *Y*${}_{t}$ [69], while removal of the exocyclic 4-amino group completely abolishes the DNAzyme activity [70]. Substitution of dA${}_{15}$ by dI only had a minor effect on activity and substitution by a δN${}^{7}$-deoxyadenine with an amino acid side-chain led to a fully active DNAzyme [67], whereas the substitution by dC reduces cleavage activity by 90% [70].

### 3.4. Non-Bridging Oxygen Atoms of the Phosphate Group between dT${}_{4}$ and dA${}_{5}$ May Be Involved in Metal Ion Coordination

**Figure 6:**
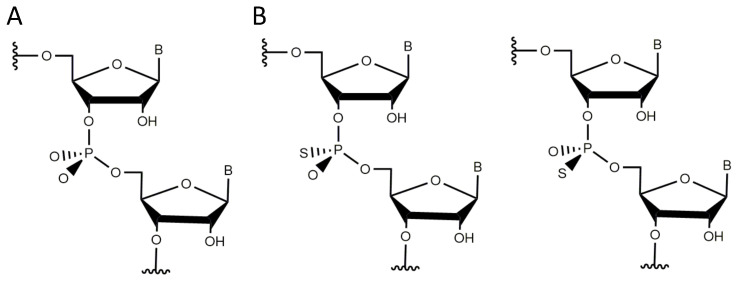
Conformations of the non-brinding oxygen atoms in a phosphodiester bond. (**A**) Prochiral phosphodiester bond between two nucleotides illustrating the absolute positions of the *pro*-*R* and *pro*-*S* non-bridging oxygens. (**B**) *S*${}_{P}$ or *R*${}_{P}$ phosphorothioate diastereomers after substitution of one of the identical non-bridging oxygen atoms by a sulfur atom.

At position dA${}_{5}$ in the DNAzyme, the exchange of the adenosine by dI significantly reduced the cleavage activity, whereas the exchange to dC only led to a twofold decrease in the activity [70]. Replacement of the nucleobase to a deoxypurine (DP) did not alter the activity compared to the unmodified DNAzyme [70]. In contrast, an abasic deoxynucleotide substitution at this position completely abolishes the function of the DNAzyme [69]. These findings strongly suggest that the exocyclic amino group at this position (position 6 of the purine ring system) is not of functional importance, but that probably the nitrogen in the ring system plays an important role in hydrogen bond formation [70]. Studies in Reference [66] revealed that also the amine at position 3 of the purine ring does not play a critical role in 10-23 DNAzyme function. Reference [65] analyzed the relevance of particular phosphates within the catalytic loop by a systematic modification of each phosphate with a phosphorothioate (PS) analogue. Within the internucleotide phosphodiesters two identical unesterified oxygen atoms that share a negative charge are attached to the sp${}^{3}$-hybridized phosphorous atom. The descriptors used for these oxygen atoms are *pro*-*R* and *pro*-*S* and they are used to distinguish between the two atoms. Substitution of one of these two non-bridging oxygen atoms by a sulfur atom leads to either the *S*${}_{P}$ or the *R*${}_{P}$ diastereoisomer (Figure 6). The activity of the modified DNAzymes can then be measured in the presence of ${\mathrm{Mg}}^{2+}$ and ${\mathrm{Mn}}^{2+}$. Reference [65] performed such an experiment in tris(hydroxymethyl)aminomethane (Tris) buffer containing 100 mM NaCl and with a 100-fold molar excess of the DNAzyme over the RNA substrate. The results of this study confirm the involvement of both non-bridging oxygens between dT${}_{4}$ and dA${}_{5}$ (referred to as d5^P^) in the hydrolysis reaction. With regard to the importance of the 6-keto group in the proximal deoxynucleotide dG${}_{6}$, Reference [65] proposes a model that involves both oxygen atoms at position d5^P^ as well as the 6-keto group of dG${}_{6}$ in the binding of one or more divalent metal ions. Experiments with thio-deoxyribozymes of stereodefined *P*-chirality also suggested that the *R*${}_{P}$ at position d9^P^ is directly involved in metal ion coordination. Since significant thio and rescue effects were also detected for the oxygen atoms at positions d2^P^, d4^P^, d10^P^, d11^P^, d12^P^, and d13^P^, these ligands may also be involved in the coordination of metal ions [65].

### 3.5. Deletion of the Deoxynucleotide dT${}_{8}$ Leads to an Active 10-23 DNAzyme

Thymidine dT${}_{8}$ was found to be the most tolerant nucleotide towards substitution by other nucleotides including dI [70], an abasic nucleotide, a C3 spacer [69] or a complete deletion [72]. It has been reported that the participation of dT${}_{8}$ in catalysis can be modulated by varying its ability to contribute to stacking interactions with 5-substituted azobenzene groups, where the specific stacking of the aromatic group with a different configuration could induce a positive or negative effect on the catalysis enhancement [78]. Introduction of a rigidly conjugated imidazolyl group at dT${}_{8}$ for expanded base stacking interaction and hydrogen-bonding network led to a slight decrease in the catalytic activity [79]. The weak base stacking interaction of the unmodified dT${}_{8}$ has a positive effect on the reaction rate. This effect has also been confirmed by studies with modified sugars, in which (*R*)- and (*S*)-2′-*C*-methyl-purine as well as locked conformations had a negative effect on the catalytic activity of the DNAzyme [71,80]. However, deletion of dT${}_{8}$ was even found to increase the cleavage activity of a DNAzyme targeting a 19-mer RNA substrate [72]. Activity assays performed with different RNA substrates showed that in all cases dT${}_{8}$ could be deleted without dramatic negative impact on the cleavage activity [72].

### 3.6. Adenine Minor Groove Interactions Play a Role in 10-23 DNAzyme-Mediated Catalysis

Mutagenesis analyses by Reference [66] with substitutions of adenosine residues within the catalytic loop by 3-deaza-adenosine (3-DA) residues reveal the importance of hydrogen bonds that arise from the N3 atoms of adenosine (dA${}_{5}$, dA${}_{9}$, dA${}_{11}$, dA${}_{12}$, dA${}_{15}$). N3 nitrogen atoms in the purine rings of guanine and adenine are known to be involved in adenine minor groove interactions (A-minor interactions), which were first discovered in ribosomal RNA [81]. Albeit 3-DA substitutions at each location decrease the activity of the 10-23 DNAzyme, dA${}_{12}$ appears to be the most affected deoxynucleotide with a decrease in $k_{\mathrm{obs}}$ of approximately 70% and of 25% in *Y*${}_{t}$. This is remarkable, since dA${}_{12}$ was reported to be not of crucial importance for the DNAzyme activity. Substitution by other naturally occurring nucleobases and N${}^{8}\Delta$N${}^{7}$-deoxyadenine analogues did not affect the activity significantly and substitution by an abasic deoxynucleotide or a C3 spacer did not decrease *Y*${}_{t}$ suggesting that dA${}_{12}$ is not important for catalysis [60,70,72].

### 3.7. A DNAzyme Variant with an 11 Nt-Containing Catalytic Loop Requires Ca${}^{2+}$ for Its Activity

To analyze the effect of DNAzyme sequence on its RNA cleavage activity, Reference [74] removed specific nucleotides from the catalytic loop region. Therefore, a reaction mixture consisting of a 200-fold molar excess of DNAzyme variant compared to RNA substrate in Tris buffer containing 25 mM divalent metal ions was used. After a reaction time of 90 min the mixture was analyzed using polyacrylamide gel electrophoresis. Their results show that a DNAzyme variant that has d(A${}_{5}$G${}_{6}$C${}_{7}$T${}_{8}$) deleted from the original sequence retains a cleavage activity of 22.2% compared to the unmodified DNAzyme in the presence of Ca${}^{2+}$. In the presence of either ${\mathrm{Mg}}^{2+}$ or ${\mathrm{Mn}}^{2+}$, the activity was reduced to 10% compared to the unmodified sequence. A DNAzyme in which the nucleotides d(A${}_{5}$G${}_{6}$C${}_{7}$T${}_{8}$) are replaced by abasic deoxynucleotides did not show any activity in the presence of either ${\mathrm{Mg}}^{2+}$, Ca${}^{2+}$, or ${\mathrm{Mn}}^{2+}$ [74]. Since a DNAzyme with abasic substitutions at positions 5-8 were not functional and the δd(A${}_{5}$G${}_{6}$C${}_{7}$T${}_{8}$) variant only shows cleavage activity in the presence of Ca${}^{2+}$, but not in the presence of ${\mathrm{Mg}}^{2+}$. The cleavage mechanism of this new subclass was proposed to be different from the original 10-23 DNAzyme that requires the presence of ${\mathrm{Mg}}^{2+}$. Ca${}^{2+}$ and ${\mathrm{Mg}}^{2+}$ do not only vary in the ionic radii and their charge density, they also prefer different coordination geometries: while Ca${}^{2+}$ forms complexes with eight ligands, ${\mathrm{Mg}}^{2+}$ prefers the formation of octahedral complexes with six ligands [82].

A summary of the effects of substitutions and modifications of single deoxynucleotides and functional groups on the activity of the 10-23 DNAzyme is given in Table 2. Mutagenesis studies by References [70] and [69] have identified functional groups that are important for DNAzyme catalysis: the exocyclic 4-amino groups of dC${}_{3}$ and dC${}_{13}$; the 6-carbonyl group of dG${}_{6}$ and dG${}_{14}$; the 2-amino group of dG${}_{6}$; the nucleobases of dG${}_{1}$, dG${}_{2}$, and dT${}_{4}$. Replacement of either position by any other naturally occurring nucleotide leads to complete loss of activity.

## 4. Influence of Metal Ions on 10-23 DNAzyme Activity

**Figure 7:**
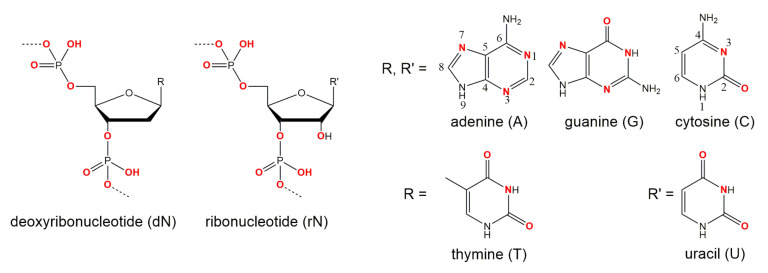
Potential metal interaction sites in DNA and RNA. The functional groups that are in principle capable of interacting with cations are highlighted in red. Besides the bridging and non-bridging oxygen atoms of the phosphate backbone of DNA and RNA and the 2′-oxygen atom in RNA sequences, the nitrogen atoms N1, N3, and N7 of purine bases, the N3 nitrogen atom of cytosine and the carbonyl oxygen atoms at position C2 and C4 in pyrimidine bases and C6 in guanosine can function as donor atoms for the coordination of metal ions at physiological pH values as well as deprotonated (N1)${}^{-}$ of guanosine and (N3)${}^{-}$ of thymidine and uracil. The exocyclic amino groups also have the potential to participate in metal ion coordination as hydrogen-bond donors within the first coordination sphere (reviewed in Reference [88]; and Reference [95]).

The role of metal ions in ribozyme-mediated catalysis [83,84,85,86,87,88,89] has been reviewed in a large number of articles, which is not the case for DNAzymes. Metal ions can either directly be involved in catalysis by forming inner-sphere contacts with functional groups of the nucleic acid, or they can play a role as cofactors without direct interaction with the nucleic acid through outer-sphere contacts. In addition, they can stabilize transition states or active conformations of a DNAzyme. Moreover, metal ions are capable of generating an electrostatic environment that changes the p*K*_a_ value or the nucleophilic character of a water molecule. They are also able to alter the properties of functional groups by polarization. $K^{+}$, ${\mathrm{Na}}^{+}$ and ${\mathrm{Mg}}^{2+}$ are the most abundant metal ions in living cells. They are associated with nucleic acids and function as metal ion cofactors for ribozymes in vivo (an excellent review article on the interaction of metal ions with nucleic acids can be found elsewhere [90]). Metal binding sites within DNA and RNA molecules are highly abundant as indicated in Figure 7. However, except the bridging and non-bridging oxygen phosphates the potential of most donor atoms to participate in metal coordination is very low due to either low or high basicity or steric hindrance, resulting in a very dynamic and unspecific binding of metal ions to nucleic acid strands (reviewed by Reference [88]). Therefore, the identification of specific metal binding sites in catalytically active nucleic acids remains a challenging task. Despite some studies on metal ion dependency of the 10-23 DNAzyme, the coordination sites and the specific roles of metal ions are poorly understood. The 10-23 DNAzyme was obtained by in vitro selection in the presence of ${\mathrm{Mg}}^{2+}$ ions [1]. Its activity strongly varies in the presence of different divalent metal cations, since the different ionic radii, the p*K*_a_ values of coordinating water molecules, and the affinity to functional groups tremendously affect the catalytic properties of the metal ion. Studies on the effect of metal ions on the DNAzyme activity have shown that divalent metal ions promote the reaction in the following order: ${\mathrm{Mn}}^{2+}$ (in 3-[4-(2-Hydroxyethyl)piperazin-1-yl]propane-1-sulfonic acid, EPPS) > Pb${}^{2+}$, Mg${}^{2+}$, Ca${}^{2+}$ > Cd${}^{2+}$ (in Tris buffer) > Sr${}^{2+}$, Ba${}^{2+}$, Zn${}^{2+}$, Co${}^{2+}$ [33]. These findings correlate with the studies of Reference [74], where the metal ions enhance the cleavage reaction in the following order: ${\mathrm{Mn}}^{2+}$ > ${\mathrm{Mg}}^{2+}$ > Ca${}^{2+}$ ≫ Ba${}^{2+}$ in Tris buffer. The variation in activity may either be due to the occupation of different binding sites within the nucleic acids as reported for either ${\mathrm{Mg}}^{2+}$ [91], ${\mathrm{Mn}}^{2+}$ [91], or Cd${}^{2+}$ [92] in the hammerhead ribozyme or the binding behavior with regard to inner- or outer-sphere contacts with the nucleic acid as reported for the same ribozyme with either ${\mathrm{Mg}}^{2+}$ [93] or ${\mathrm{Mn}}^{2+}$ [94]. For all studies performed with ${\mathrm{Mn}}^{2+}$ at pH 7.5 one has to keep in mind that the actual ${\mathrm{Mn}}^{2+}$ concentration might be overestimated due to oxidation of the metal at pH 7.5 [33].

Reference [96] compared the observed activity of the 10-23 DNAzyme depending on the p*K*_a_ value of different metal ions and they found that the logarithm of the *k*_obs_ value was inversely proportional to the p*K*_a_ value. Folding of the DNAzyme in the presence of different ${\mathrm{Mg}}^{2+}$ concentrations has been analyzed using circular dichroism (CD) and Förster resonance energy transfer (FRET) in low ionic strength buffer, revealing three different folding events at ${\mathrm{Mg}}^{2+}$ concentrations of 0.5 mM (compact structure of the DNAzyme), 5 mM (proper positioning of binding arms to bind RNA), and 15 mM (completely organized catalytic domain) [97]. The authors concluded that binding of ${\mathrm{Mg}}^{2+}$ to the 10-23 DNAzyme induces significant rearrangement of the catalytic loop leading to optimal folding of the catalyst. Besides what is known for small ribozymes [98,99], in DNAzyme-mediated catalysis divalent metal ions cannot fully be replaced by monovalent metal ions. Moreover, our group recently found that the cleavage reaction of the 10-23 DNAzyme is drastically reduced at high ionic strength (${\mathrm{Na}}^{+}$ or $K^{+}$) in the presence of ${\mathrm{Mg}}^{2+}$.

Reference [44] investigated different 10-23 DNAzyme complexes with the RNA substrate in 2 ns MD simulations in the presence and absence of different metal ions. Controversially, in the presence of monovalent $K^{+}$ ions, the DNAzyme:RNA complex tends to form a typical B-type helix. This is surprising, since DNA:RNA duplexes are usually known to form A-type helices [64]. While the sugar pucker in the RNA strand of the duplex varies between 3′-endo (here termed *North*-type ) and 2′-endo (here termed *South*-type) conformation, the DNA residues of the binding arms and the catalytic loop have the sugar moieties in the 2′-endo conformation, as in B-type DNA. In the presence of ${\mathrm{Mg}}^{2+}$ cations the complex is not only more stabilized, but also shows a significantly different structure compared to the one in the presence of only $K^{+}$ ions. ${\mathrm{Mg}}^{2+}$ ions lead to a significant change in the folding of the catalytic loop, that is no longer directed away from the DNA:RNA duplex, as found in the presence of $K^{+}$, but bent towards the DNA:RNA duplex, with a stretch of the catalytic domain being located close to the cleavage site. While in the presence of $K^{+}$, the sugar moieties of the flanking DNA strands predominantly show *South*-endo conformation, in the presence of ${\mathrm{Mg}}^{2+}$ *North*-endo conformation predominates. Comparing the structures of the native and mutated DNAzymes derived from MD simulations, Reference [44] found that the presence of ${\mathrm{Mg}}^{2+}$ induces the formation of an electrostatic pocket by the catalytic loop and that the cleavage reaction may be assisted by three ${\mathrm{Mg}}^{2+}$ cations. Their findings are in agreement with a study by Reference [11], which suggests a cooperative binding of three ${\mathrm{Mg}}^{2+}$. Reference [44] reported that several cations are placed near the scissile phosphate and retained there during the simulation. However, the authors conclude that their model cannot explain the catalytic activity and proposed that a conformational change must happen prior to the cleavage reaction. A coarse-grained Brownian dynamics simulation shows that the 10-23 DNAzyme bends its substrate away from the cleavage site, exposing the reactive site and buckling the DNAzyme catalytic loop [100]. Reference [32] has shown that—in general—metal ions may operate in the catalytic transesterification of the phosphodiester bonds in RNA substrates according to four main strategies that they termed: α-catalysis (facilitation of a proper geometric alignment for “in-line” nucleophilic attack), β-catalysis (charge neutralization on the non-bridging oxygens of the scissile phosphate bond), γ-catalysis (metal-assisted deprotonation of the reactive 2′-hydroxyl) and δ-catalysis (stabilization of the 5′-oxygen of the leaving group). The cleavage reaction of the 10-23 DNAzyme has also been studied with regard to the catalytic strategy [32]. A combinatorial analysis of ${\mathrm{Mg}}^{2+}$ and pH dependency reveals that the catalytic strategy of this DNAzyme exceeds the exclusive use of the combinatorial catalysis of α- [101] and γ-catalysis [102], since the rate constant reached under suboptimal conditions exceeds the combined αγ-speed limit [32,33]. However, since no thio effect was observed if the non-bridging phosphate oxygen at the cleavage site was replaced by a sulfur atom [103], it is likely that the 10-23 DNAzyme does not use metal coordination to such an oxygen atom for rate enhancement, excluding β-catalysis [104] as an additional strategy for the cleavage reaction [32].

## 5. Critical DNAzyme Positions and Their Potential Involvement in DNAzyme Catalysis

**Figure 8:**
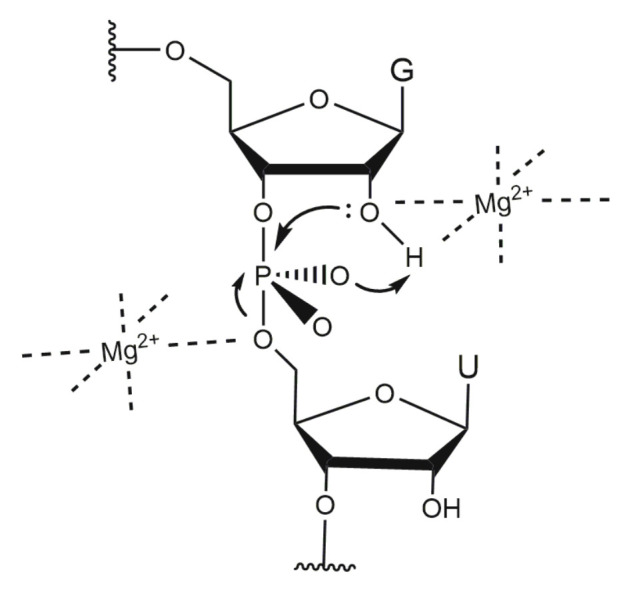
Proposed mechanistic model for the metal ion-mediated RNA cleavage catalysed by a 10-23 DNAzyme Two metal ions (here ${\mathrm{Mg}}^{2+}$) are involved in a triester-like mechanism, that has originally been proposed for the RNA-cleavage reaction catalysed by a hammerhead ribozyme [105]. One metal ion acts as a Lewis acid and coordinates to the 2′-OH group. Thereby, the OH bond is polarized and migration of the proton to the *R*${}_{P}$ oxygen is facilitated. The second metal ion coordinates to the 5′-oxygen leaving group and neutralizes the negative charge during the cleaveage of the P-O bond. Based on Reference [103].

The exhaustive characterization of different 10-23 DNAzyme mutants and chemical modifications provide insights into the impact of various deoxynucleotides and functional groups on DNAzyme catalysis, even in the absence of high-resolution structural information. The mutagenesis studies by References [60,70] clearly demonstrate the relevance of the exocyclic 4-amino group in dC${}_{3}$ and dC${}_{13}$ as well as the 6-keto group in dG${}_{3}$. At position dG${}_{14}$, the 2-amino group and the 6-keto group play an important role in DNAzyme catalysis. In addition, the free electron pairs of N1 or N7 at position 5 are crucial for the RNA cleavage activity. The effect of substitutions of deoxynucleotides by abasic deoxynucleotides or C3 spacers that add a high degree of flexibility to the DNAzyme loop also reveal that structural pre-arrangement of some of the 2′-deoxyribose moieties is of importance for the correct function of the 10-23 DNAzyme [69]. The most striking observations have been made for dT${}_{8}$, which can be deleted without significantly decreasing the DNAzyme activity. Introduction of 2′-(*R*)-CH${}_{3}$ and 2′-(*S*)-CH${}_{3}$ modified purine nucleosides leads to a decrease in RNA cleavage activity. However, the reduction in activity was higher when the 2′-(*R*)-CH${}_{3}$ derivative was introduced [80]. The activity was even more reduced when locked nucleic acid building blocks were introduced at position 8, suggesting that a certain degree of conformational flexibility at this position is crucial [71]. Position 8 is also of interest with regard to metal ion coordination. Based on results from cleavage assays with phosphorothioate substitutions at the non-bridging phosphate oxygen atoms analyzing the thio and rescue effects in the presence of ${\mathrm{Mn}}^{2+}$ and ${\mathrm{Mg}}^{2+}$ it is reasonable to assume that the *R*${}_{P}$ oxygen at position d9^P^ is directly involved in metal ion coordination [65]. Based on the Pearson acid-base concept [77], a ${\mathrm{Mg}}^{2+}$ ion has a lower affinity to the polarizable sulfur-containing DNAzyme resulting in a lower cleavage rate. A recovery of the cleavage rate in the presence of ${\mathrm{Mn}}^{2+}$ instead of ${\mathrm{Mg}}^{2+}$ is called rescue effect. In addition to phosphorothioate substitutions within the catalytic loop region, the effect of phosphorothioate substitution at the scissile phosphate in the RNA substrate has been investigated [96,103]. Here, it was found that the cleavage of the *S*${}_{P}$ thio analog was only slightly reduced compared to the cleavage of the unmodified RNA substrate in the presence of ${\mathrm{Mg}}^{2+}$ ions. In contrast, the *R*${}_{P}$ phosphorothioate could not be cleaved, even in the presence of high ${\mathrm{Mg}}^{2+}$ concentrations (100 mM). However, this thio effect could not be rescued in the presence of ${\mathrm{Mn}}^{2+}$, suggesting that there is no direct coordination of a metal ion to the non-bridging oxygen atoms at the scissile phosphodiester bond. This hypothesis is in agreement with earlier suggestions of References [32,96]. Summarizing the mechanistic results by References [32,33,103], one can exclude β-catalysis as well as the exclusive use of α- and γ-catalysis. A catalytic mechanism, which is in agreement with the data of Reference [32] and Reference [103], proposes two metal ions that both bind to the scissile site of the RNA substrate: the first metal ion acts as a Lewis acid that directly coordinates to the 2′-oxygen atom and hereby promotes the deprotonation of the 2′-hydroxyl group (γ-catalysis). The proton then migrates to the *R*${}_{P}$ oxygen of the scissile phosphate. A second metal ion is coordinated to the 5′-oxygen of the leaving group to neutralize the negative charge during the cleavage of the P-O bond (δ-catalysis) (Figure 8).

## 6. Conflicting Results

Although the influence of deoxynucleotide substitutions and modifications, deoxyribose and phosphate modifications within the catalytic loop and the binding arms of the 10-23 DNAzyme, as well as its tolerance towards different metal ions and pH conditions have been extensively studied, the mechanism by which the DNAzyme cleaves its specific RNA substrate as well as the role of different functional groups is only poorly understood. The main cause is arguably the absence of satisfactory structural data of the 10-23 DNAzyme. However, one major obstacle when it comes to the interpretation of different mutational studies is the low level of comparability of the experimental setup. While some experiments were designed to determine only the yield after a given period of time (*Y*${}_{t}$) of the cleaved substrate, others were designed to determine rate constants ($k_{\mathrm{obs}}$). Furthermore, experiments vary in ${\mathrm{Mg}}^{2+}$ concentrations used to facilitate DNA-mediated RNA cleavage or the nature of the substrate: while some experiments were performed in the presence of *all*-RNA substrates other experiments were carried out using chimeric DNA/RNA substrates, with only the nucleotide at the cleavage site being a ribonucleotide. Also, DNAzyme and RNA substrate design can have a massive influence on the kinetic behavior of the DNAzyme, since the length and sequence dramatically influence the dissociation constants of the DNAzyme:RNA complex. In addition, temperature, monovalent cation levels as well as the type of reaction buffer can have an impact on DNAzyme activity. In Table 3, we summarize the experimental conditions used in the most important studies on DNAzyme activity.

## 7. Conclusions

Efforts to elucidate the reaction mechanism are essential for attempts to use DNAzymes for therapeutic applications. Structural information on DNAzymes is scarce, therefore, we rely on functional data for insights into DNAzyme catalysis. The data summarized in this review provides interesting insights into the role of the different regions of the 10-23 DNAzyme. The binding arms of the DNAzyme are responsible for substrate recognition and form a double helical structure, while the catalytic loop forms an unusual three-dimensional conformation. Taking a closer look at the cleavage site, we conclude that the neighboring nucleotides of the scissile A or G in the RNA and DNA strand play an important role in catalysis. All mutations and modifications at these positions indicate a flexibility or equilibrium of rN${}_{-1}$–dA${}_{15}$ and rN${}_{+1}$–dG${}_{1}$. These nucleotides might act as hinges between the catalytic loop and the DNA-RNA arms. They allow the rigid catalytic loop to move against the rigid double helices and adopt a particular conformation that enable the cleavage reaction.

Studies focusing on the residues within the catalytic loop have shown that the exocyclic groups of the nucleotides dG${}_{14}$ and dG${}_{6}$ are essential for DNAzyme function. Deletion of the 6-keto group in either dG${}_{14}$ or dG${}_{6}$ leads to a dramatic decrease in catalytic activity, while the substitution of the oxygen atom of the keto-group by sulfur results in a decreased reaction rate in the presence of ${\mathrm{Mg}}^{2+}$. The activity can be rescued in the presence of ${\mathrm{Mn}}^{2+}$, suggesting the interaction of this atom with the divalent metal ion during hydrolysis. Deletion of the 2-amino group in dG${}_{6}$ also reduces the activity of the 10-23 DNAzyme, suggesting that these deoxynucleotides play an important role in the cleavage of RNA catalyzed by the 10-23 DNAzyme.

The challenge remains to understand the mechanism of 10-23 DNAzyme catalysis and the role of different functional groups in the DNAzyme sequence. We believe that three aspects are crucial for future studies. First, studies using comparable experimental setups and conditions would be beneficial for the research field. This includes determining rate constants instead of yields and using RNA substrates instead of chimeric substrates. In addition, the effects of buffers and ionic strength should be investigated thoroughly, to ensure that effects observed at different conditions are not a result of the reaction conditions. Second, starting conditions of DNAzyme reactions should be chosen in respect of the desired output. For example, a condition with preformed, buffer-equilibrated 1:1 DNA:RNA complexes and reaction initiation via addition of divalent metal ions should be used to investigate effects of the divalent ions on single-turnover reaction rates, while excess of substrate is essential to characterize the Michaelis-Menten parameters of DNAzyme catalysis. Finally, a high-resolution structure of the 10-23 DNAzyme is necessary to obtain detailed insights into the spatial arrangement of the DNAzyme in complex with its RNA substrate. A major challenge is the formation of irrelevant conformations due to the palindromic sequence. This challenge can be overcome by using variants of the DNAzyme with single nucleotides exchanged in the catalytic loop. Some of these variants retain a considerable level of activity, but the mutation leads to a sequence that is not palindromic and, thus, should not form the biological irrelevant duplex conformation. Another attempt to stabilize the catalytically active form of the complex is to use DNA- or RNA-binding proteins that prohibit duplex formation. Alternatively, methods not relying on crystallization, including NMR and EPR spectroscopy could provide valuable insights into molecular design and dynamics of 10-23 DNAzymes when in complex with a target RNA fragment. Initial experimentally validated structural models will also be essential as reliable starting structure for in silico techniques that may provide more detailed mechanistic insights into the different states of the catalysis process.

Taken together, we have summarized molecular features of the 10-23 DNAzyme in this review in order to contribute to a better understanding of these fascinating biocatalysts. Our detailed analysis reveals important aspects to be considered in future attempts to elucidate the structure of DNAzymes in complex with the RNA substrate and unravel the mechanism of the reaction catalyzed by the 10-23 DNAzyme.

## Figures and Tables

**Table 1:** Effect of nucleotide substitutions at position +1 of the DNAzyme (dN${}_{+1}$) and the sequence of the nucleotides at the cleavage site of the RNA substrate (paired as well as unpaired N${}_{+1}$) on the RNA cleavage rate. DAP${}_{+1}$, 2,6-diaminodeoxypurine; dδN${}^{7}$A${}_{+1}$, 7-deazadeoxyadenine; d(N${}^{8}\Delta$N${}^{7}$)A${}_{+1}$, 8-aza-7-deazadeoxyadenine; for formulas see Figure 3.

A${}^{↓}$U${}_{+1}$	=	G${}^{↓}$U${}_{+1}$	≥	G${}^{↓}$C${}_{+1}$	≫	A${}^{↓}$C${}_{+1}$
R${}^{↓}$U${}_{+1}$		R${}^{↓}$C${}_{+1}$				
|	>	|				
dA${}_{+1}$		dG${}_{+1}$				
R${}^{↓}$Y${}_{+1}$		R${}^{↓}$Y${}_{+1}$		A${}^{↓}$C${}_{+1}$		A${}^{↓}$C${}_{+1}$
|	>	|		|	≫	|
dI${}_{+1}$		dR${}_{+1}$		dI${}_{+1}$		dG${}_{+1}$
A${}^{↓}$U${}_{+1}$		A${}^{↓}$U${}_{+1}$		G${}^{↓}$U${}_{+1}$		G${}^{↓}$U${}_{+1}$
|	>	|		|	≈	|
dA${}_{+1}$		DAP${}_{+1}$		dA${}_{+1}$		DAP${}_{+1}$
R${}^{↓}$U${}_{+1}$		R${}^{↓}$U${}_{+1}$			R${}^{↓}$U${}_{+1}$	
|	≈	|	≈		|	
dA${}_{+1}$		dδN${}^{7}$A${}_{+1}$		d(N${}^{8}\Delta$N${}^{7}$)A${}_{+1}$		

**Table 2:** Effect of selected substitutions of single deoxynucleotides on the activity (*Y_t_* and *k*_obs_) of the 10-23 DNAzyme towards short RNA substrates. Parameters were reported for reactions performed in the presence of tenfold excess [70,72] or in the presence of 100-fold excess of the DNAzyme over the RNA substrate [65], and in the presence of 100- to 200-fold excess of the DNAzyme over a chimeric DNA/RNA substrate [66,69]. Unless specified differently, the values refer to $Y_{t}$. If values refer to $k_{\mathrm{obs}}$ this is explicitly mentioned. “–” indicates that this modification was not investigated on this position, “x” indicates that no yield $Y_{t}$ could be measured for this modification. All values are given in percentages compared to the substrate cleavage performed by the unmodified DNAzyme; that is, $k_{\mathrm{obs}}=100\;\cdot \;k_{\mathrm{obs},\;\mathrm{mutant}}/k_{\mathrm{obs},\;\mathrm{wild}\text{-}\mathrm{type}}$ and $Y=100\;\cdot \;Y_{t,\;\mathrm{mutant}}/Y_{t,\;\mathrm{wild}\text{-}\mathrm{type}}$.

dN	G ^a^	A ^a^	T ^a^	C ^a^	I ^a^	Abasic ^b^	Spacer ^b^	Deletion ^c^	2-AP ^a^	DP ^a^	3-DA ^d^	s${}^{6}$G ^e^	δN${}^{7}$G ^e^
G${}_{1}$	–	<5 ^f^	<5 ^f^	<5 ^f^	40 ^f^	106 ^g^ $k_{\mathrm{obs}}=65$	100 ^g^ $k_{\mathrm{obs}}=43$	<10 ^f^	–	–	–	–	–
G${}_{2}$	–	<5 ^f^	<5 ^f^	<5 ^f^	60 ^f^	97 ^g^ $k_{\mathrm{obs}}=15$	88 ^h^ $k_{\mathrm{obs}}=19$	<10 ^f^	–	–	–	–	–
C${}_{3}$	10 ^f^	70 ^f^	20 ^f^	–	<5 ^f^	100 ^g^ $k_{\mathrm{obs}}=54$	103 ^g^ $k_{\mathrm{obs}}=40$	10 ^f^	–	–	–	–	–
T${}_{4}$	<5 ^f^	<5 ^f^	–	5 ^f^	<5 ^f^	73 ^h^ $k_{\mathrm{obs}}=7$	75 ^h^ $k_{\mathrm{obs}}=6$	<5 ^f^	–	–	–	–	–
A${}_{5}$	<5 ^f^	–	5 ^f^	55 ^f^	10 ^f^	6 ^h^ $k_{\mathrm{obs}}=2$	–	<5 ^f^	–	105 ^f^	100 ^g^ $k_{\mathrm{obs}}=50$	–	–
G${}_{6}$	–	<5 ^f^	<5 ^f^	<5 ^f^	100 ^f^	x	x	<6 ^f^	–	–	–	$k_{\mathrm{obs}}<5$	$k_{\mathrm{obs}}<1$
C${}_{7}$	20 ^f^	75 ^f^	60${}^{a}$	–	20 ^f^	79 ^h^ $k_{\mathrm{obs}}=7$	61 ^h^ $k_{\mathrm{obs}}=9$	78 ^f^	–	–	–	–	–
T${}_{8}$	90 ^f^	90 ^f^	–	75 ^f^	110 ^f^	106 ^g^ $k_{\mathrm{obs}}=124$	109 ^g^ $k_{\mathrm{obs}}=129$	105 ^f^	–	–	–	–	–
A${}_{9}$	40 ^f^	–	90 ^f^	10 ^f^	50 ^f^	71 ^h^ $k_{\mathrm{obs}}=6$	69 ^h^ $k_{\mathrm{obs}}=5$	30 ^f^	–	–	98 ^g^ $k_{\mathrm{obs}}=49$	–	–
C${}_{10}$	60 ^f^	75 ^f^	60 ^f^	–	60 ^f^	47 ^h^ $k_{\mathrm{obs}}=4$	58 ^h^ $k_{\mathrm{obs}}=4$	15 ^f^	–	–	–	–	–
A${}_{11}$	55 ^f^	–	85 ^f^	30 ^f^	75 ^f^	94 ^h^ $k_{\mathrm{obs}}=9$	94 ^h^ $k_{\mathrm{obs}}=10$	30 ^f^	–	–	101 ^g^ $k_{\mathrm{obs}}=31$	–	–
A${}_{12}$	35 ^f^	–	85 ^f^	75 ^f^	95 ^f^	97 ^h^ $k_{\mathrm{obs}}=12$	101 ^h^ $k_{\mathrm{obs}}=11$	30 ^f^	–	–	74 ^g^ $k_{\mathrm{obs}}=17$	–	–
C${}_{13}$	5 ^f^	50 ^f^	10 ^f^	–	10 ^f^	x	x	<5 ^f^	–	–	–	–	–
G${}_{14}$	–	<5 ^f^	<5 ^f^	<5 ^f^	10 ^f^	x	x	<5 ^f^	5 ^f^	–	–	–	–
A${}_{15}$	70 ^f^	–	50 ^f^	10 ^f^	90 ^f^	84 ^h^ $k_{\mathrm{obs}}=9$	86 ^h^ $k_{\mathrm{obs}}=11$	<5 ^f^	–	–	103 ^g^ $k_{\mathrm{obs}}=32$	–	–

[a] according to [70]; [b] according to [69]; [c] according to [72]; [d] according to [66]; [e] according to [65]; [f] given as $Y_{t=20\min}$; [g] given as $Y_{t=120\min}$; [h] given as $Y_{t=24\;h}$.

**Table 3:** Conditions used in selected important kinetic studies on the 10-23 DNAzyme with short substrates in vitro.

Reference	Substrate	Length of Binding Arms	Mg${}^{2+}$ [mM]	NaCl [mM]	RNA	DNAzyme	Duration and Temperature	Output
[17]	RNA	7/7	10	150	0.2 µM	0.5–500 nM	various timepoints, 37 °C	$k_{\mathrm{obs}}$, single and multiple turnover
[50]	RNA	variable	10	–	0.04 µM	0.32 µM	various timepoints, 37 °C	$k_{\mathrm{obs}}$, single turnover
[56]	RNA	9/9	10	–	0.6 µM	5 µM	various timepoints, up to 1 h; 37 °C	$k_{\mathrm{obs}}$, single turnover
[96]	RNA	7/7	25	–	0.06–0.1 µM	0.5–10 µM	various timepoints, 37 °C	$k_{\mathrm{cat}}$, multiple turnover
[60]	DNA/w RNA	9/9	6 (2)	–	0.02 µM (0.2 µM)	2 µM (0.02 µM)	various timepoints, 37 °C	$k_{\mathrm{obs}}$, single turnover (multiple turnover)
[68]	DNA/w RNA	9/9	0.5–10	–	0.02 µM	2 µM	various timepoints, 37 °C	$k_{\mathrm{obs}}$, single turnover
[106]	DNA/w RNA	9/9	20	–	0.02 µM	2 µM	various timepoints, 37 °C	$k_{\mathrm{obs}}$, single turnover
[79]	DNA/w RNA	9/9	2.18	–	0.02 µM	2 µM	various timepoints, 37 °C	$k_{\mathrm{obs}}$, single turnover
[65]	DNA /w RNA	8/8	3	100	0.1 µM	10 µM	various timepoints, 37 °C	$k_{\mathrm{obs}}$, single turnover
[103]	DNA/w RNA	8/8	0.02	100	0.1 µM	10 µM	various timepoints, 37 °C	$k_{\mathrm{obs}}$, single turnover
[73]	RNA	6/8	25	–	0.01–1 µM	0.001 µM	various timepoints, 37 °C	$k_{\mathrm{cat}}$, multiple turnover
[80]	RNA	8/8	0.5–10	–	0.5 µM	5 µM	various timepoints, 37 °C	$k_{\mathrm{obs}}$, single turnover
[71]	RNA	8/8	0.5–10	–	0.5 µM	0.5 µM	various timepoints, 37 °C	$k_{\mathrm{obs}}$, single turnover
[1]	RNA	8/8	variable	variable	variable	variable	various timepoints, 37 °C	$k_{\mathrm{obs}}$ and $k_{\mathrm{cat}}$, single and multiple turnover
[33]	RNA	8/8	2	150	variable	variable	various timepoints, 37 °C	$k_{\mathrm{obs}}$ and $k_{\mathrm{cat}}$, single and multiple turnover
[63]	RNA	9/9	10	100	1 µM	0.01 µM	20 min, 37 °C	initial velocity, multiple turnover
[74]	RNA	8/6	25	–	5 nM (tenfold excess)	1 µM (n.a.)	various timepoints, 37 °C	*Y*${}_{t}$ ($k_{\mathrm{cat}}$), single turnover (multiple turnover)
[11]	RNA	9/9	0.1-1	100	0.1 µM	0.1 µM	various timepoints, 37 °C	$k_{\mathrm{cat}}$, single turnover
[69]	DNA/w RNA	9/9	500	200	0.005 µM	0.5 µM	various timepoints, 37 °C	*Y*${}_{t}$ and $k_{\mathrm{obs}}$, single turnover
[61]	DNA/w RNA	9/9	2	–	0.02 µM	2 µM	various timepoints, –	*Y*${}_{t}$ and $k_{\mathrm{obs}}$, single turnover
[70]	RNA (RNA)	9/9	10	–	0.1 µM	1 µM	20 min, 37 °C	*Y*${}_{t}$, single turnover
[72]	RNA (RNA)	9/9 (9/9)	10 (10)	– (–)	1 pmol (0.01–1 µM)	10 pmol (0.001 µM)	20 min (various timepoints), 37 °C	*Y*${}_{t}$ ($k_{\mathrm{cat}}$, multiple turnover)
[57]	DNA/w RNA	9/9	2	–	n.a.	100-fold excess	Various timepoints, 37 °C	$k_{\mathrm{obs}}$, single turnover
[59]	DNA/w RNA	9/9	2	–	0.002 µM	2 µM	various timepoints, 37 °C	$k_{\mathrm{obs}}$, single turnover
